# Analgesic Effect of Dexamethasone after Arthroscopic Knee Surgery: A Randomized Controlled Trial

**DOI:** 10.1155/2016/4216469

**Published:** 2016-10-04

**Authors:** Jairo Moyano, Maria García, Maria Caicedo

**Affiliations:** Department of Anesthesiology, Hospital Fundación Santa Fe de Bogotá, Carrera 7 No. 117-15, Bogotá, Colombia

## Abstract

*Background.* Dexamethasone is sometimes used as a coanalgesic because of its anti-inflammatory properties.* Objective*. To evaluate opioid use, postoperative pain intensity, and side effects after a single dose of dexamethasone in patients undergoing arthroscopic knee surgery.* Methods*. In this randomized controlled study patients were randomized to receive either 10 mg of intravenous dexamethasone (DM group) or 0.9% normal saline (NS group) during the intraoperative period. Primary outcomes were pain intensity and total morphine and codeine use after surgery.* Results*. Seventy-eight patients were included in the study. The DM group showed statistically significant higher pain intensity at the fourth postoperative hour (DM: 3.96/10, standard deviation [SD] 0.54; NS: 2.46/10, SD 0.45; *p* = 0.036). No statistically significant difference in total opioid use (morphine plus codeine) was identified with 15.9 (SD 1.97) codeine tablets used in DM group and 20 (SD 2.14) in NS group (*p* = 0.25).* Discussion*. Pain intensity tended to decrease in both groups suggesting morphine as the main source of analgesia.* Conclusions*. Intravenous dexamethasone during the intraoperative period has no clinical impact on postoperative pain intensity during the first 48 h after arthroscopic knee surgery. This trial is registered with R000020892.

## 1. Introduction

Tissue lesions formed during surgery induce the production of multiple nociceptive mediators such as prostaglandins, which are produced via the arachidonic acid cascade. Glucocorticoids decrease the level of prostaglandin synthesis by indirectly inhibiting the activity of phospholipase A2 and cyclooxygenase II. Glucocorticoids can also modulate the inflammatory response by inhibiting the production of cytokines, including tumor necrosis factor *α*, interleukin-1*β*, interleukin-6, C-reactive protein, and leukocyte receptors. For this reason, glucocorticoids can be used in the treatment of acute pain [1–4].

Two meta-analyses conclude there is a minimum but statistically significant analgesic benefit when administering a single intravenous perioperative dose of dexamethasone [5, 6]. However, they do not discriminate results by type of surgery but give conclusions regarding different surgeries in general. Previous studies have determined the efficacy of adjunctive glucocorticoid administration in reducing pain after orthopedic procedures [7, 8]. Uncontrolled postoperative pain can affect patients' functional recovery and comfort, both critical factors for outpatients who expect to resume their daily activities as soon as possible. Accordingly, current recommendations indicate that multimodal treatment approaches should be used for acute pain occurring after knee surgery to achieve better results [9, 10]. However, patients still experience severe pain after knee surgery [7].

Although optimal analgesia is the primary desired outcome of postoperative pain management, reducing the incidence of adverse effects is also important, particularly in outpatients. Combining treatments with different mechanisms of action allows clinicians to use lower doses of each agent, thereby limiting the adverse effects associated with each. For example, a combination of nonsteroidal anti-inflammatory drugs, opioids, and peripheral nerve blocks with local anesthetics has proven to be effective in controlling inflammation and acute pain [10].

While dexamethasone may play a role in multimodal pain management therapy, more studies are needed before its routine use can be recommended [10–12]. The aim of this study was to determine whether the intraoperative use of dexamethasone reduces postoperative pain intensity and analgesic use in patients undergoing arthroscopic knee surgery.

## 2. Methods

This study had a randomized, blinded, placebo-controlled, parallel group design and was conducted at a university hospital with 190 beds. It was approved by the institutional ethics committee and all procedures were in accordance with the Declaration of Helsinki. We included 78 outpatients aged 18 years or older who were scheduled to undergo arthroscopic knee surgery by one of the surgeons. Exclusion criteria were cognitive impairment or communication difficulties, a high risk of postoperative nausea and vomiting, and contraindications to dexamethasone/diclofenac use. Patients were randomly assigned to control or treatment groups according to a computer-generated table. This randomization sequence was then given to the pharmacy coordinator (the only person to know the sequence); group assignment remained unknown to those directly involved in patient care.

The pharmacy staff members present in the operating rooms were responsible for preparation of the study solutions, which were labeled for group 0 or group 1. Immediately before anesthetic induction, the syringe with the appropriate study solution was given to the anesthesiologist in charge of the patient. The assignation sequence was sealed in an envelope kept at the hospital pharmacy, and a copy was maintained at the Department of Anesthesiology office until the end of the study. The content of the syringes remained unknown to the anesthesiologist, the patients, and the anesthesia staff who collected the data.

In the control group (NS group), patients received 2 mL of 0.9% normal saline intravenously at the onset of anesthetic induction. In the treatment group (DM group), patients received 2 mL of a 5 mg/mL dexamethasone phosphate solution at the onset of anesthetic induction; the two study solutions were physically indistinguishable. Patients underwent standard anesthetic protocols: 2-3 mg of midazolam was administered intravenously at the start of induction, followed by 3–5 mg/kg of thiopental sodium, 0.05–0.1 mcg of remifentanil·kg^−1^·min^−1^, and inhaled sevoflurane at a minimum alveolar concentration of 0.5–1 (1-2% of sevoflurane) and FiO_2_ of 0.7 was maintained. All patients received 50 mg of diclofenac intravenously immediately after intubation and 0.1 mg/kg of morphine intravenously approximately 20 min before the end of surgery.

We assessed pain intensity using a visual analog scale (VAS); adverse effects, such as nausea or vomiting, were recorded in the postanesthesia care unit at 4, 8, 12, and 24 h postoperatively. Upon discharge, each patient received an envelope containing 30 tablets of 30 mg codeine phosphate; patients were instructed to use up to 10 tablets daily (300 mg) and to record their daily intake in a booklet. Patients were then called and questioned regarding the total number of codeine tablets consumed within 48 h postoperatively.

### 2.1. Statistical Analysis

To confirm an advantage of dexamethasone over placebo, assuming a 30% reduction in pain intensity from hospital admission to 48 h after surgery, we calculated that a sample size of 80 patients (40 patients per group) would be needed given a *β* statistical power of 80% and an expected patient withdrawal rate of 10%.

Patients' characteristics were recorded in an Excel database. Continuous variables were described using measures of central tendency and dispersion. STATA version 10.1 was used for statistical analysis. Student's *t*-test was used to evaluate differences in mean values between the groups and to assess correlations regarding morphine use. A Wilcoxon rank sum test was performed to evaluate differences in patients' satisfaction with codeine analgesic treatment between the groups and to assess correlations between codeine and morphine use. Analysis of variance was used to compare repeated variables between the groups. Statistical significance was set as *p* < 0.05.

## 3. Results

**Figure 1:**
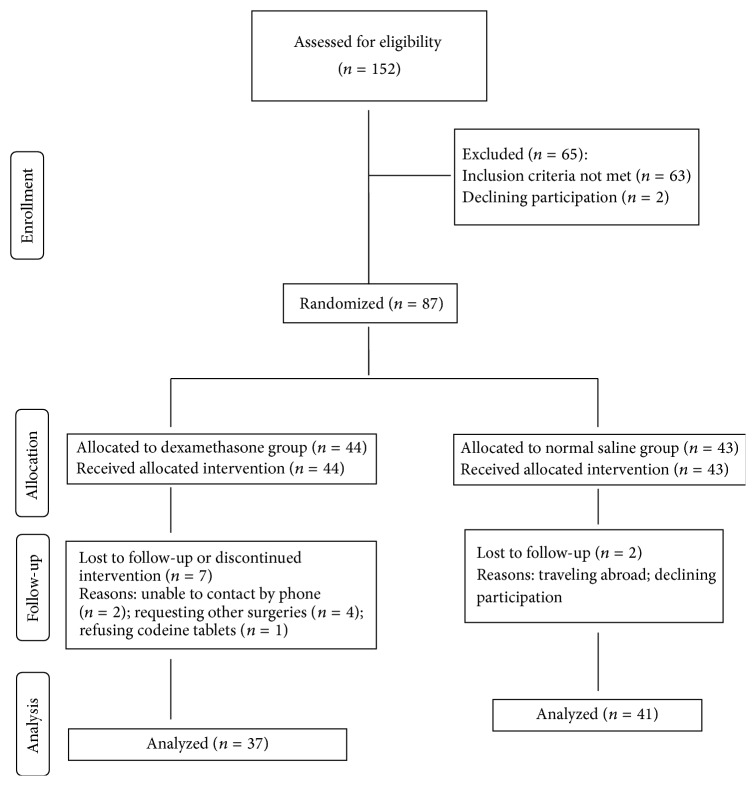
Study flow chart.

A total of 152 patients were screened; 65 of these patients were withdrawn during enrollment because they did not meet inclusion criteria or declined to participate. Eighty-seven patients were included in the study (expecting a patient withdrawal rate of 10%), and 78 (90%) of them (37 and 41 in the DM and NS groups, resp.) completed the study (Figure 1). A total of 7 patients in the DM group and 2 patients in the NS patients were lost. Patients' demographic characteristics, types of surgical procedures, and surgical duration were similar in the treatment and placebo groups (Table 1). In both groups, most of the procedures were performed in under 2 h. However, a statistically significant difference in the number of patients lost to follow-up was observed between the groups (*p* = 0.015).

### 3.1. Intensity of Pain

**Figure 2:**
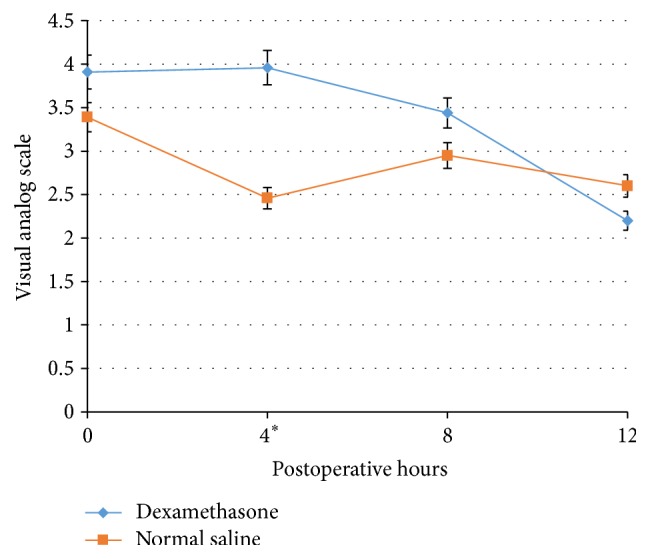
Pain intensity by visual analog scale (VAS) during the study period ^*∗*^ *p* < 0.05. The asterisk indicates that only at the 4th hour was the value considered statistically significant.

Overall, VAS scores tended to decrease in both the DM and NS groups across time (Figure 2). There were no statistical differences in the VAS scores at different postoperative time points except for the fourth hour; the DM group exhibited a higher VAS score than the NS group at this time point (DM, 3.96/10; NS, 2.46/10; 95% confidence interval [CI] 0.096–2.91; *p* = 0.0366) (Table 2).

### 3.2. Use of Morphine

As shown in Table 3, the total dose (milligrams) of morphine administered during the intraoperative and postoperative periods was higher in the NS group than in the DM group, but this difference was not statistically significant (95% CI, −2.2 to 1.70; *p* = 0.79). The mean morphine dose administered during the postoperative period was lower in the DM group, but again the difference was not statistically significant. No correlation was identified between the doses administered during the intraoperative and postoperative periods.

### 3.3. Use of Codeine

In both treatment groups, the lowest consumption of codeine tablets occurred on the day of surgery; consumption increased on the first postoperative day and then decreased again on the second postoperative day. The mean number and total number of codeine tablets taken during the first 48 h postoperatively were lower in the DM group (Table 4), although this difference was not statistically significant (*p* = 0.2557).

No correlation was observed between the number of codeine tablets taken and the morphine dose administered at the different time points evaluated. No significant differences in secondary outcomes, that is, patients' average level of satisfaction with analgesic treatment (*p* = 0.69) and the presence of adverse effects, were observed between the groups (data not shown).

## 4. Discussion

In the setting of multimodal analgesia with NSAID and systemic morphine the addition of intravenous dexamethasone does not appear to add any significant additional analgesia for knee arthroscopy patients. We accept the null hypothesis regarding the analgesic effect of dexamethasone during the early postoperative period after arthroscopic knee surgery. There were no decreases in postoperative opioid analgesic use among the patients in the DM group during their time in the postanesthesia care unit or during the following 2 days. This finding is in accordance with a previous study involving surgical patients who developed somatic or visceral pain; there was no difference in pain intensity scores between the dexamethasone and placebo group in this previous study [13]. The same study found that opioid analgesic use during the first 24 h after surgery was less among patients who received 15 mg of dexamethasone systemically but did not differ among those receiving lower doses. This finding may partly explain the lack of a significant difference in opioid use between the groups in our study, given that patients in the DM group received a 10 mg dose of dexamethasone.

Pain intensity tended to decrease in both groups and reached a low level (mild pain) in the first 12 h postoperatively. The lack of a significant difference in pain intensity between the groups suggests that morphine was the main source of analgesia. The finding of similar pain intensity between the groups in our study conflicts with reports showing a significant decrease in pain following steroid administration.

Our study's most important findings regarding pain intensity were the low initial scores in both treatment groups that persisted throughout the study period and the lack of a significant difference in opioid analgesic use between the groups. The low pain intensity scores may have been responsible for the absence of intergroup differences; previous studies have reported mild postoperative pain intensity [14]. It should be noted that there was a trend towards lower total analgesic use (morphine and codeine) in the DM group. No significant differences were observed in the occurrence of adverse effects or in patients' satisfaction with analgesic treatment.

Therefore, this study fails to show additional benefit of adding intravenous dexamethasone. A study evaluating the endocrine and immune effects of dexamethasone in total knee replacement found it inhibited the increase in monocyte count and had an effect on cortisol concentrations regulating the extent of systemic inflammation [4]. However, it is well known that pain is considered a subjective experience, and different techniques by different surgeons could have an impact on the degree of trauma. These might explain why our study fails to show any benefit on VAS scores. Two meta-analysis demonstrating benefits of dexamethasone included studies on patients undergoing general anesthesia and were found to be minimal although statistically significant but they draw no conclusions on orthopedic surgery and, more specifically, on knee surgery [5, 6].

The main limitation of this study was its small sample size, which diminished the statistical power and made it difficult to identify differences between the groups. However, given that no significant differences were found between the groups at baseline, the chance of making a type I error was small. The short postoperative follow-up period could be considered another limitation. However, we found no differences in pain intensity or analgesic use during the critical postoperative period when patients returned home; accordingly, we did not expect large differences in analgesic use or increases in acute pain intensity after this period. Another limitation was having surgeries done by various surgeons instead of one since we did not measure degree of trauma on each patient nor did we describe the technique used by each surgeon which could have influenced pain level. One last limitation was measuring VAS score at rest only and not again under dynamic conditions to evaluate how pain changes with ambulation.

Based on our results, administering 10 mg intravenous dexamethasone during the intraoperative period does not improve analgesia on postoperative pain intensity during the first 48 h after arthroscopic knee surgery when administered in conjunction with another anti-inflammatory. Morphine and codeine use, treatment satisfaction, and the incidence of adverse effects were similar in the treatment and control groups. Further studies using higher doses and an opioid different to codeine are advised.

## Figures and Tables

**Table 1:** Patient characteristics.

	Number of patients (%)			
	DM group (*n* = 37)	NS group (*n* = 41)	Total (*n* = 78)	*p* values for comparisons between the groups
Sex				
Male	27 (73%)	29 (70.7%)	56 (71.8%)	0.705
Female	10 (27%)	12 (29.3%)	22 (28.2%)	0.376

Mean age, years	39.9	44.3	42.1	0.194

Surgical procedure				
ACL repair	11 (29.8%)	16 (39%)	27 (34.6%)	0.109
Meniscectomy	9 (24.3%)	10 (24.4%)	19 (24.4%)	0.746
Synovectomy	1 (2.7%)	2 (4.9%)	3 (3.84%)	0.414
Multiple	12 (32.4%)	10 (24.4%)	22 (28.2%)	0.546
Other	4 (10.8%)	3 (7.3%)	7 (8.97%)	0.593

Surgical time, hours				
<2	21 (56.8%)	24 (58.6%)	45 (58%)	0.404
2 to 3	14 (37.8%)	16 (39%)	30 (38%)	0.606
≥3	2 (5.4%)	1 (2.4%)	3 (4%)	0.414

Number lost to follow-up	7 (18.9%)	1 (2.4%)	8 (10%)	0.015

ACL, anterior cruciate ligament; DM, dexamethasone; NS, normal saline.

**Table 2:** VAS scores for pain intensity during the first 12 h postoperatively.

Postoperative time, hours	0 (PACU)	4	8	12
DM group				
Number of patients	35	32	32	30
Mean	3.91	3.96	3.44	2.2
SD	0.49	0.54	0.48	0.42

NS group				
Number of patients	41	41	41	41
Mean	3.39	2.46	2.95	2.6
SD	0.41	0.45	0.46	0.41

Mean difference	0.524	1.5	0.4816	−0.4

95% CI	(−0.75, 1.18)	(0.096, 2.91)	(−0.85, 1.83)	(−1.62, 0.8)

*p* value	0.413	0.037	0.472	0.503

CI, confidence interval; DM, dexamethasone; NS, normal saline; PACU, postanesthesia care unit; SD, standard deviation; VAS, visual analog scale.

**Table 3:** Total morphine dose (combined intraoperative and postoperative doses).

	Total morphine dose (mg)
DM group (*n* = 37)	
Mean	6.40
SD	0.79

NS group (*n* = 41)	
Mean	6.66
SD	0.60

Mean difference	−0.253

95% CI	(−2.2, 1.70)

*p *value	0.80

CI, confidence interval; DM, dexamethasone; NS, normal saline; SD, standard deviation.

**Table 4:** Total number of codeine tablets taken during the first 48 h postoperatively.

	Number of codeine tablets
DM group (*n* = 37)	
Mean	15.9
SD	1.97

NS group (*n* = 41)	
Mean	20
SD	2.14

Mean difference	−4.16

*p* value	0.256

DM, dexamethasone; NS, normal saline; SD, standard deviation.

## Additional Points

Glucocorticoids, such as dexamethasone, are sometimes used in the treatment of acute pain after orthopedic procedures. We sought to determine if intraoperative use of dexamethasone reduces postoperative pain intensity and analgesic use in patients undergoing arthroscopic knee surgery. We found that administering intravenous dexamethasone during the intraoperative period has no clinical impact on postoperative pain intensity during the first 48 h after arthroscopic knee surgery.
